# Anti-Inflammatory Activity of an In Vitro Digested Anthocyanin-Rich Extract on Intestinal Epithelial Cells Exposed to TNF-α

**DOI:** 10.3390/molecules27175368

**Published:** 2022-08-23

**Authors:** Antonio Speciale, Romina Bashllari, Claudia Muscarà, Maria Sofia Molonia, Antonella Saija, Shikha Saha, Peter J. Wilde, Francesco Cimino

**Affiliations:** 1 Department of Chemical, Biological, Pharmaceutical and Environmental Sciences, University of Messina, Viale F. Stagno D’Alcontres 31, 98166 Messina, Italy; 2 “Prof. Antonio Imbesi” Foundation, University of Messina, 98100 Messina, Italy; 3 Food Innovation and Health Programme, Quadram Institute Bioscience, Norwich Research Park, Norwich NR4 7UQ, UK

**Keywords:** inflammatory bowel disease, anthocyanins, inflammation, NF-κB, adaptative cellular response, in vitro digestion, biostability

## Abstract

Background: The consumption of foods rich in anthocyanins (ACN) have been associated with beneficial properties in chronic inflammatory disorders such as intestinal bowel diseases (IBD). These effects were attributed not only to a direct antioxidant mechanism but also to the modulation of cell redox-dependent signaling. However, ACN bioavailability is low for their poor stability in the digestive tract, so ACN gastrointestinal digestion should be considered. Methods: To have a more realistic knowledge of the effects of ACN, we performed an in vitro simulated gastrointestinal digestion of an ACN-rich purified and standardized bilberry and blackcurrant extract (BBE), followed by an evaluation of ACN composition modification (HPLC-DAD and pH differential method) and antioxidant activity (FRAP assay). Then, we studied the effects of BBE gastrointestinal extract on Caco-2 exposed to TNF-α. Results: The results confirmed the high instability of ACN in the mild alkaline environment of the small intestine (17% recovery index). However, the digested BBE maintained part of its bioactivity. Additionally, BBE gastrointestinal extract inhibited the TNF-α-induced NF-κB pathway in Caco-2 and activated the Nrf2 pathway. Conclusions: Although ACN stability is affected by gastrointestinal digestion, the anti-inflammatory and antioxidant activity of digested extracts were confirmed; thus, the loss of ACN can probably be counterweighed by their metabolites. Then, ACN introduced by diet or food supplements could represent an approach for IBD prevention.

## 1. Introduction

Inflammatory bowel diseases (IBD) are a group of multifactorial inflammatory pathologies characterized by the alternation of acute and remission phases [1] and by a notable infiltration of leukocytes in the intestine, which, in addition to proinflammatory cytokines, produce huge amounts of reactive oxygen species (ROS) [2]. The overproduction of ROS, together with a reduction in antioxidant activity, is also referred to as oxidative stress, and this condition has been associated with increased intestinal permeability, impaired immune response, and damage to proteins and nucleic acids [2,3]. One of the mechanisms by which oxidative stress initiates and propagates intestinal inflammation is mediated by the NF-κB pathway. Although the redox-sensitive nuclear transcription factor NF-κB in the intestine is involved in maintaining intestinal epithelial cell homeostasis and modulating intestinal permeability [4], the chronic activation of NF-κB is typical of IBD and may play an important role in the exacerbation of inflammation of the intestinal epithelium [5,6,7].

Conventional therapies for the treatment of IBD involve the use of anti-inflammatory agents and corticosteroids that often have side effects [2]. A valid alternative to the use of anti-inflammatory drugs is represented by immunomodulators which, however, possess cytotoxic effects which consequently can increase other pathologies and infections [8,9].

Recently, the use of natural or synthetic antioxidants able to modulate the pathway of NF-κB could represent a new, useful, and efficient complementary approach for IBD [10,11]. In particular, there has been a strong and growing interest in the study of anthocyanins (ACN), a subclass of polyphenols that are widely present in fruit and vegetables. ACNs are soluble pigments found in nature as glycosides of the flavilium salt (2-phenylbenzopyryl) and differ from each other by the number of hydroxyl groups and their degree of methylation, and the nature and quantity of sugar bound to the molecule [12]. So far, 17 of their aglycones, known as anthocyanidins, have been identified, and seven of them are widely found in nature. Although there are only 17 anthocyanidins, more than 600 ACNs exist in the plant kingdom.

Recent studies indicate that ACNs have health-promoting and disease-preventing properties. In particular, they have proven to be effective in many inflammatory-associated diseases such as cardiovascular disease, diabetes, obesity, inflammatory bowel disease, and cancer, thanks to their antioxidant and anti-inflammatory properties [13]. ACNs are beneficial to weight loss, reducing inflammation, and promoting energy consumption. Additionally, they protect the blood vessels in cardiometabolic disease by enhancing the production of vasodilating factors such as nitric oxide and decreasing inflammation and oxidative stress. Furthermore, many in vivo and in vitro studies support the beneficial effects of ACNs in various other chronic inflammatory diseases, such as IBD [14,15,16,17]. In particular, they act not only as antioxidants but also by modulating cell redox-dependent signaling. In vitro studies reported that ACNs are able to induce the Nuclear factor-erythroid 2 related factor 2/Antioxidant response element (Nrf2/ARE) pathway, which plays a key role in maintaining redox homeostasis in the intestinal mucosa by regulating the expression of detoxifying and antioxidant enzymes [14,18,19,20,21]. This mechanism seems to be critical for the inhibition of proinflammatory pathways such as the NF-κB one. In fact, possible crosstalk has been proposed between the Nrf2 and NF-κB pathway [22,23] since Nrf2 activation induces the degradation by ubiquitination of IKKβ, a critical regulatory kinase in the NF-κB activation and nuclear translocation [24].

However, many mechanistic studies of ACN in intestinal inflammation are usually based on the in vitro evaluation of crude extracts or pure compounds. ACN bioavailability seems to be low since they are susceptible to degradation due to factors such as pH, temperature, light, and enzymes [25]. In an acidic environment (pH < 3), they occur in their most stable form, as flavylium cations; otherwise, at pH > 4, they exist in the form of carbinol and chalcone, and the latter can undergo further chemical modifications, finally producing phenolic compounds of degradation [26]. Moreover, prior to passage into the bloodstream, ACNs undergo biotransformation by the gut microbiota and then by the enterocytes metabolizing enzymes producing a wide range of byproducts and glucurono-, sulfo-, or methyl-derivatives. ACN glycosides can be hydrolyzed to the aglycone forms at the mucosal brush border membrane prior to absorption similar to many other flavonoids [27]. In this form, the hydrolyzed ACN aglycon can enter the epithelial cells by passive diffusion as a result of its increased lipophilicity and its proximity to the enterocyte membrane. Additionally, in the large intestine, the ingested ACNs are metabolized by the gut microbiota to a range of low-molecular-weight catabolites, mainly phenolic acids and other phenols descending from the B-ring of the ACN skeleton [26].

In light of this, the effects of gastrointestinal digestion of ACNs should be taken into consideration to understand the real impact of daily intake on health protection and improvement. Recently, the in vitro gastrointestinal simulation of digestion represented a valid experimental model to evaluate the changes in the stability of phytochemicals and the effect on the biological activity of pure or crude extracts.

For a more realistic assessment of the positive effects of ACNs, it is important to know their fate and stability following gastrointestinal digestion; in this work, an in vitro simulated gastrointestinal digestion was performed on a standardized extract of blackcurrant and bilberry rich in ACNs (BBE). Our research then focused on the changes in ACN composition and antioxidant activity following simulated digestion and the effects of the intestinal phase of simulated digestion in an in vitro model of intestinal inflammation using Caco-2 cells monolayers exposed to TNF-α. In particular, we evaluated the anti-inflammatory and antioxidant effects by focusing on the NF-κB proinflammatory pathway and the activation of an antioxidant response modulated by the Nrf2 pathway.

## 2. Results

### 2.1. Effects of Gastrointestinal Digestion on Anthocyanins Recovery

**Figure 1:**
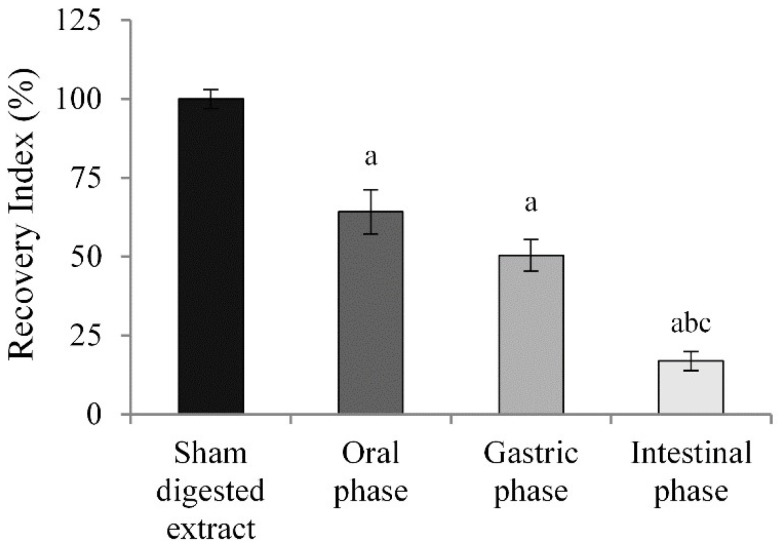
**Recovery Index (RI) of anthocyanins.** The total ACN content (TAC) in sham-digested and BBE digested extracts (all diluted in parallel to 8 mL) was analyzed by the pH differential method, expressed as C3G equivalents, and finally, RI was calculated. The sham-digested extract was used as reference for the RI calculation. Results are expressed as mean ± SD calculated from three independent experiments. ^a^ *p* < 0.05 vs. sham-digested; ^b^ *p* < 0.05 vs. oral phase ^c^ *p* < 0.05 vs. gastric phase.

It is well known that ACNs exert numerous beneficial effects on health thanks to their antioxidant, anti-inflammatory, antiproliferative, and anti-angiogenic activities [21]. However, ACNs are highly unstable substances. During the physiological process of digestion, they are subjected to pH and enzymatic metabolization-induced modifications that influence their antioxidant activity [28]; in vitro simulated digestion models have been developed in recent years, which are essential to evaluate the stability, bioavailability, and bioactivity of numerous phytochemicals [29,30,31,32]. Therefore, to evaluate the recovery and hence the stability of ACN after gastrointestinal digestion, a static in vitro simulated digestion of BBE was performed. The total ACN content (TAC) was analyzed on both sham-digested extract and the samples obtained during the digestion phases, using a differential pH method, and finally, the recovery index (RI) of digesta vs. sham-digested sample was calculated. The data in Figure 1 show that after oral digestion, a decrease in ACN content of 35.8% compared to the sham-digested extract was observed, probably due to the neutral environment (pH 7) and to their transformation into carbinol, which is an unstable and colorless form. Following simulated gastric digestion with pepsin and in an acid environment, the ACN content, although not in a statistically significant manner, was reduced by 13.8% compared to the oral phase sample with an RI of 50.4%. These findings suggest that the low pH value in the stomach mainly contributes to the high stability of these compounds, which at this pH occur in the chemical structure of a stable flavylium cation. Finally, further simulated intestinal digestion with pancreatin in a neutral environment strongly influenced the RI of can, resulting in a statistically significant reduction compared to the oral and/or gastric phase, with an RI value of 16.9%. This considerable reduction in the RI value in the intestinal phase is probably due to the transformation of ACN into carbinol and/or their degradation products [26]. Our results are consistent with other studies in which a significant reduction in berry ACNs after gastrointestinal digestion was highlighted [33,34].

### 2.2. Effect of In Vitro Simulated Digestion on the Antioxidant Activity of BBE

**Figure 2:**
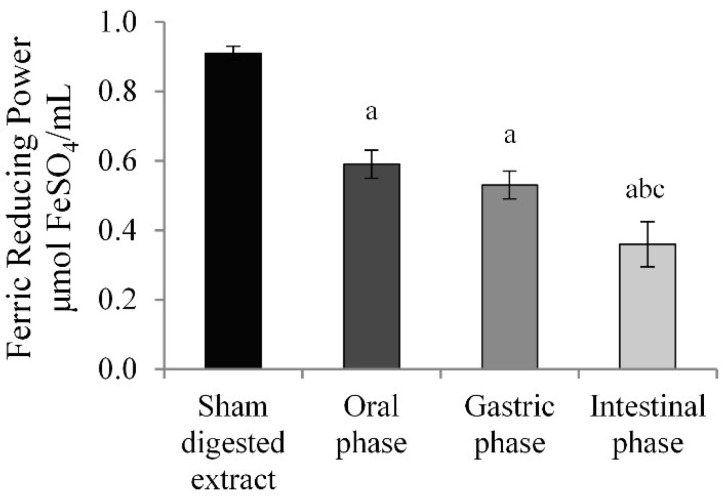
**FRAP assay.** Results are reported as μmol FeSO_4_/mL of sham-digested or digested extract and expressed as mean ± SD of three independent experiments.^a^ *p* < 0.05 vs. sham-digested extract; ^b^ *p* < 0.05 vs. oral phase ^c^ *p* < 0.05 vs. gastric phase.

ACNs are well known for their high antioxidant capacity [35]. During gastrointestinal digestion, ACNs undergo chemical changes, and these can lead to alterations of the antioxidant capacity [26]. Therefore, the variation of the antioxidant power of the ACNs contained in BBE during gastrointestinal digestion was evaluated. The FRAP test was performed on sham or digested extracts, a commonly used method for measuring the in vitro total antioxidant activity [36]. The data shown in Figure 2 highlighted a significant reduction in the antioxidant power (35%) in the oral digested compared to the undigested extract, and this value was slightly reduced (42% vs. sham-digested extract) after the gastric phase. Finally, a further loss of 18% was observed after the intestinal digestion compared to the gastric digested extract and a 60% vs. the sham-digested extract. These data are consistent with those of other in vitro studies where a similar reduction in antioxidant power was observed by analyzing currants, apples, and strawberries after gastrointestinal digestion [31,32,37].

The comparison between the results of the TAC and FRAP assay evidences a parallel decrease of the ACN content and the antioxidant power of the sham-digested extract and the oral and gastric digested extracts. This suggests that the antioxidant capacity of the BBE extract is mainly attributable to the ACN content. In contrast, comparing intestinal vs. gastric digestion, the considerable reduction in ACN content following simulated intestinal digestion corresponded only to a lower decrease in the antioxidant power, suggesting that the degradation products, such as phenolic compounds, formed during gastrointestinal digestion, could also be involved in the antioxidant activity. Therefore, the loss in ACN concentration due to gastrointestinal digestion may be balanced by the formation of new metabolites with antioxidant activity.

### 2.3. Characterization of BBE after In Vitro Simulated Gastrointestinal Digestion

**Figure 3:**
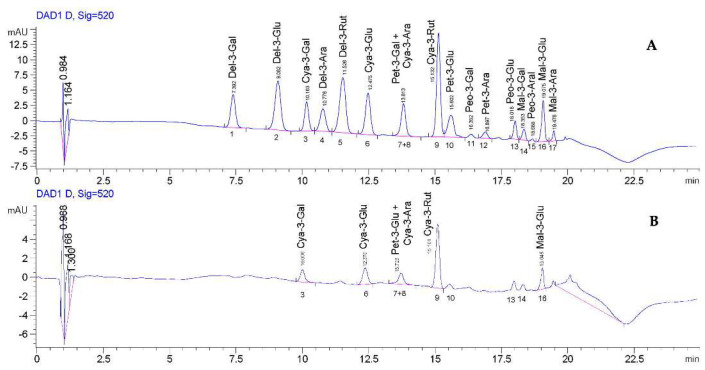
**HPLC–DAD representative chromatograms (see the text for details).** Anthocyanin profile of sham-digested extract (**A**) and intestinal digested phase (**B**). The chromatograms were acquired at 520 nm. Peaks: **1**–*Delphinidin–3–galactoside*; **2**–*Delphinidin–3–glucoside*; **3**–*Cyanidin–3–galactoside*; **4**–*Delphinidin–3–arabinoside*; **5**–*Delphinidin–3–rutinoside*; **6**–*Cyanidin–3–glucoside*; **7 + 8** *Cyanidin–3–arabinoside + Petunidin–3–galactoside*; **9**–*Cyanidin–3–Rutinoside*; **10**–*Petunidin–3–glucoside*; **11**–*Peonidin–3–galactoside*; **12**–*Petunidin–3–arabinoside*; **13**–*Peonidin–3–glucoside*; **14**–*Malvidin–3–galactoside*; **15**–*Peonidin–3–arabinoside*; **16**–*Malvidin–3–glucoside*; **17**–*Malvidin–3–arabinoside*.

To evaluate the stability of the individual ACNs after gastrointestinal digestion, a HPLC-DAD analysis was conducted on the sham-digested and intestinal-digested phases of BBE. The analysis confirmed the presence of 17 ACN in the sham-digested extract. The most abundant compounds were delphinidin and cyanidin glycosides. Especially, cyanidin-3-rutinoside and delphinidin-3-rutinoside were the predominant ACN, followed by delphinidin-3-glucoside and C3G. Furthermore, smaller amounts of glycosides of peonidin, petunidin, and malvidin were also found. As noted previously (Figure 1), the total ACN content in the digested intestinal extract was significantly reduced. Of the 17 ACN present in the undigested extract, only six of them were detected in the intestinal digested phase, and a significant reduction in the content of the remaining ACN was observed (Figure 3). The presence of hydroxyl groups in the ortho position of the B ring makes the ACN more unstable and susceptible to oxidation, and a reduction in their stability increases with the number of hydroxyl groups [38]. In addition, the presence of methoxy substituents in the B ring, present in malvidin, slightly protects it from degradation [39,40]. Taking this into account, delphinidin, having as substituents two hydroxyl groups in the B ring, is the most unstable ACN among those determined. In fact, the glycosides of delphinidin, which were the most abundant compounds in the undigested extract, were not detected in the intestinal digested extract. These findings agree with those of the study by Lucas-Gonzalez and coworkers [30], since, by examining the content of maqui berry containing derivatives of cyanidin and delphinidin, only two derivatives of cyanidin after intestinal digestion were identified. Similarly, Pérez-Vicente and coworkers [41] showed that all the ACN contained in a sweet pomegranate juice decreased after in vitro intestinal digestion with a more marked reduction for the derivatives of delphinidin than for those of cyanidin. As reported in Table 1, the order of the recovery index of ACN determined in the digested intestinal extract was as follows: cyanidin-3-galactoside (29.97 %) > cyanidin-3-rutoside (29.42%) > malvidin-3-glucoside (26.53%) > cyanidin-3-arabinoside + petunidin-3-galactoside (25.83%) > C3G (21.83%). Although the glycosidic derivatives of cyanidin, having one less hydroxyl group than the delphinidin derivatives, are more unstable compounds than malvidin, peonidin, and petunidin derivatives, they have proved to be the most abundant compounds in the intestinal digested extract probably due to their higher initial content in BBE.

### 2.4. Protective Effect of Digested BBE on TNF-α-Induced Intestinal Inflammation

**Figure 4:**
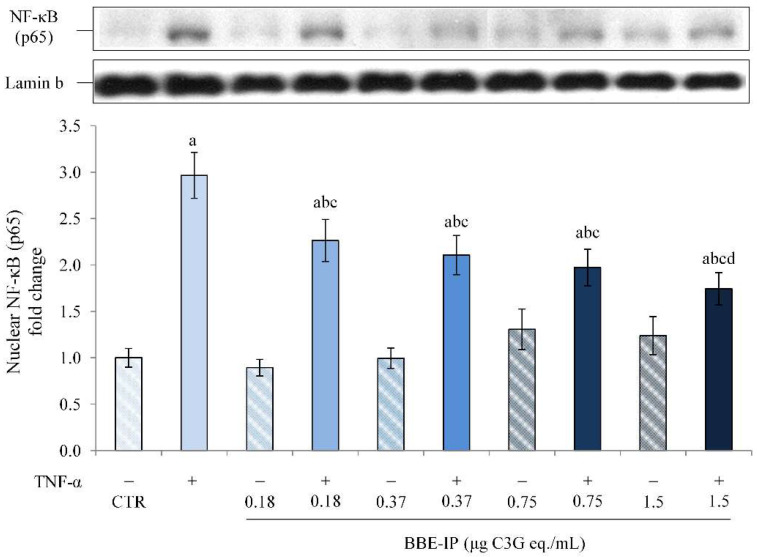
**Nuclear NF-κB (p65).** Differentiated Caco-2 cells were pretreated or not for 24 h with different concentrations of the BBE-IP (0.18, 0.37, 0.75, 1.5 μg C3G eq./mL). Cells were subsequently exposed to 50 ng/mL TNF-α for 6 h. Cells treated with the vehicles alone were used as controls (CTR). Results are reported as fold change against CTR and expressed as mean ± SD of three independent experiments. p65 values were normalized to the corresponding Lamin b value. ^a^ *p* < 0.05 vs. CTR; ^b^ *p* < 0.05 vs. TNF-α; ^c^ *p* < 0.05 vs. same BBE-IP dose unexposed to TNF-α; ^d^ *p* < 0.05 vs. BBE-IP 0.18 exposed to TNF-α.

In order to understand the protective effect of the ACNs contained in BBE after gastrointestinal digestion against intestinal inflammation, a widely validated in vitro model of the inflamed intestinal epithelium was used to study intestinal inflammatory mechanisms [18,42,43,44,45]. This experimental model consists of the use of Caco-2 cell monolayers and TNF-α as an inflammation-inducing agent. Most of the in vitro studies evaluate the mechanisms of action of ACN considering concentrations (>50 µM) that would seem to be too high to reach the target site in physiological conditions. Therefore, our goal was to evaluate the molecular mechanisms underlying the activity of ACN by considering concentrations that can be easily reached in the intestine after intestinal digestion. For this aim, the digested extract of BBE (BBE-IP), i.e., the extract subjected to all phases of digestion still rich in ACN, was used in our experiments. The monolayers of Caco-2 cells were apically pretreated for 24 h with different concentrations of BBE-IP (0.18, 0.37, 0.75, and 1.5 µg/mL calculated with the differential pH method and expressed as C3G eq.) and subsequently exposed to TNF-α 50 ng/mL for 6 h. One has to underline that the highest concentration used in experiments on Caco-2 cells (1.5 μg/mL C3G eq.), taking into account the value of 16.9% of intestinal recovery (Figure 1), corresponds almost to 20 μM C3G of an undigested extract, and this concentration has been used in several in vitro studies to evaluate the anti-inflammatory effects of ACN [18,46,47,48]. At the end of the treatments, the activation of the main sensitive redox transcription factor involved in inflammatory processes, NF-κB, was evaluated [49]. As shown in Figure 4, in our experimental conditions, elevated nuclear levels of the p65 NF-κB subunit were observed in cells exposed to TNF-α. Pretreatment with BBE-IP dose-dependently counteracted TNF-α-induced nuclear translocation of p65, with the highest concentration tested that almost halved TNF-α-effect. Furthermore, BBE-IP alone had no effect on basal nuclear p65 levels.

**Figure 5:**
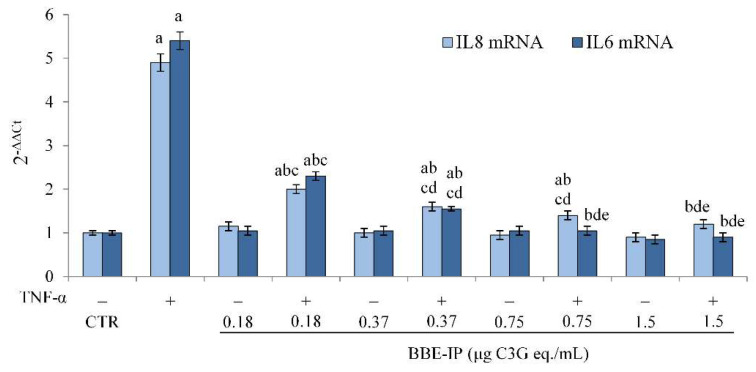
**IL-8 and IL-6 mRNA expression.** Differentiated Caco-2 cells were pretreated or not for 24 h with different concentrations of the BBE-IP (0.18, 0.37, 0.75, 1.5 μg C3G eq./mL). Cells were subsequently exposed to 50 ng/mL TNF-α for 6 h. Cells treated with the vehicles alone were used as controls (CTR). IL-6 and IL-8 mRNA expressions were analyzed by real time PCR and data are expressed as 2^−ΔΔCt^ and normalized to CTR. 18S rRNA was used as housekeeping gene; ^a^ *p* < 0.05 vs. CTR; ^b^ *p* < 0.05 vs. TNF-α; ^c^ *p* < 0.05 vs. all BBE-IP concentrations not exposed to TNF-α; ^d^ *p* < 0.05 vs. BBE-IP 0.18 μg C3G eq./mL + TNF-α; ^e^ *p* < 0.05 vs. BBE-IP 0.37 μg C3G eq./mL + TNF-α.

The activation of NF-κB induced by various pro-inflammatory agents leads to a rapid modulation of the transcription of genes that code for pro-inflammatory cytokines and immunoregulatory mediators, such as IL-8 and IL-6 [50]. Therefore, in order to confirm the transcriptional activity of NF-κB, the gene expression of IL-8 and IL-6 was evaluated. Our data confirmed that TNF-α is able to induce the activation of the inflammatory process in Caco-2 cells, as demonstrated by the increase in IL-8 and IL-6 mRNA levels (Figure 5). On the other hand, this increase was significantly reduced by the pretreatment with BBE-IP at all tested concentrations and in a dose-dependent manner. In this case, the highest concentration tested was able to restore IL-8 mRNA levels to those of control cells. Furthermore, the IL-6 mRNA levels were similar to the control cells starting from a BBE-IP concentration as low as 0.75 μg C3G eq./mL. BBE-IP alone had no effect on both IL-8 and IL-6 mRNA basal levels.

These data confirm the ability of the intestinal digested extract to prevent the inflammation induced by TNF-α. This effect, observed at very low ACN concentrations (0.18 µg C3G eq./mL), is very likely due to the presence of these pigments in the digested extract. However, we cannot exclude the involvement and a synergistic effect of ACN metabolites/degradation products formed during gastrointestinal digestion.

### 2.5. Digested BBE Affect Nrf2/Keap1 Pathway Activation

**Figure 6:**
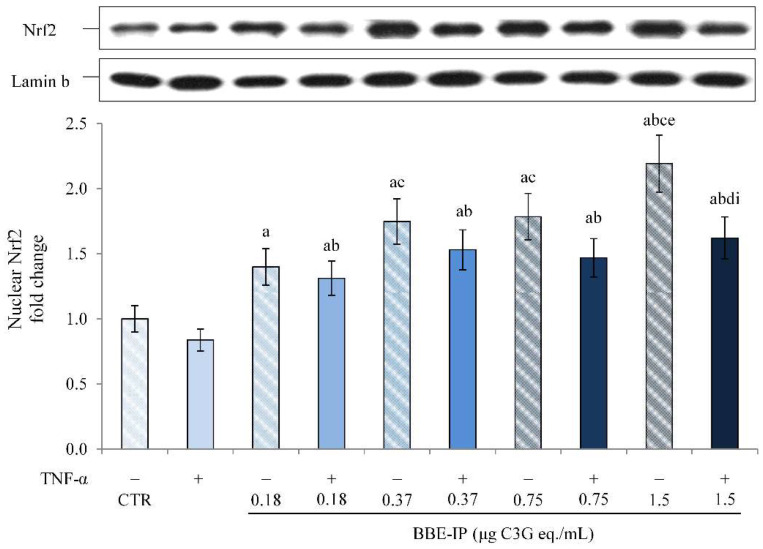
**Nuclear Nrf2.** Differentiated Caco-2 cells were pretreated or not for 24 h with different concentrations of the BBE-IP (0.18, 0.37, 0.75, 1.5 μg C3G eq./mL). Cells were subsequently exposed to 50 ng/mL TNF-α for 6 h. Cells treated with the vehicles alone were used as controls (CTR). Results are reported as fold change against CTR and expressed as mean ± SD of three independent experiments. Nrf2 values were normalized to the corresponding Lamin b value. ^a^ *p* < 0.05 vs. CTR; ^b^ *p* < 0.05 vs. TNF-α; ^c^ *p* < 0.05 vs. BBE-IP 0.18 unexposed to TNF-α; ^d^ *p* < 0.05 vs. BBE-IP 0.18 exposed to TNF-α; ^e^ *p* < 0.05 BBE-IP 0.37 unexposed to TNF-α; ^i^ *p* < 0.05 BBE-IP 1.5 unexposed to TNF-α.

It has been shown that ACNs exert an indirect antioxidant activity through the induction of an adaptive cellular response involving the activation of the Nrf2/Keap1 pathway [21]. Under normal conditions, the Nrf2 transcription factor resides in the cytoplasm bound with its inhibitor Keap1. Otherwise, in conditions of stress, Nrf2 is released by its inhibitor; it translocates to the nucleus, where it binds to the antioxidant response elements (AREs), leading to the activation of the transcription of genes that code for antioxidant and detoxifying proteins [51]. In order to elucidate the mechanisms underlying the positive effects of BBE-IP, the nuclear localization of Nrf2, and its transcriptional activity, were evaluated. As previously demonstrated [18], TNF-α slightly reduced Nrf2 activation, although not significantly (Figure 6). In contrast, pretreatment with BBE-IP dose-dependently increased the nuclear levels of Nrf2 both in cells exposed or not to TNF-α. However, TNF-α slightly reduced BBE-IP effects on Nrf2 nuclear translocation. 

**Figure 7:**
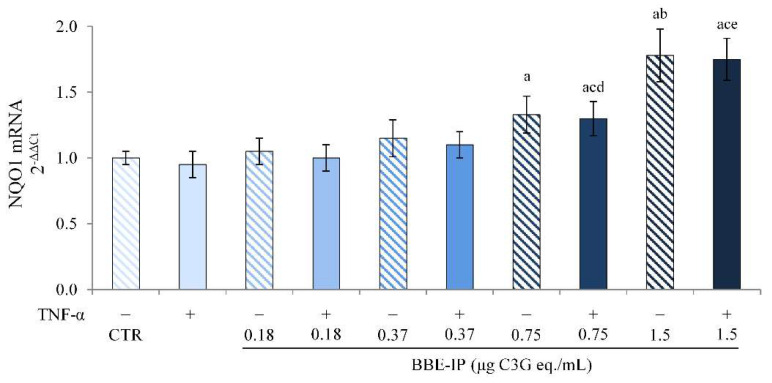
**NQO1 mRNA expression.** Differentiated Caco-2 cells were pretreated or not for 24 h with different concentrations of the BBE-IP (0.18, 0.37, 0.75, 1.5 μg C3G eq./mL). Cells were subsequently exposed to 50 ng/mL TNF-α for 6 h. Cells treated with the vehicles alone were used as controls (CTR). NQO-1 mRNA expression was analyzed by real time PCR and data are expressed as 2^−ΔΔCt^ and normalized to CTR. 18S rRNA was used as housekeeping gene. ^a^ *p* < 0.05 vs. CTR; ^b^ *p* < 0.05 vs. all BBE-IP concentrations not exposed to TNF-α; ^c^ *p* < 0.05 vs. TNF-α; ^d^ *p* < 0.05 vs. BBE-IP 0.18 μg C3G eq./mL; ^e^ *p* < 0.05 vs. all BBE-IP concentrations exposed to TNF-α.

Additionally, the transcriptional activity of Nrf2 was confirmed by determining the gene expression of NQO1, a gene that presents an ARE sequence. The data in Figure 7 show that TNF-α had no effect on NQO1 levels, as also previously reported [18]. The pretreatment with BBP-IP induced the overexpression of NQO1 mRNA starting from the concentration of 0.75 μg C3G eq./mL, both in cells exposed or not to TNF-α.

These data support the hypothesis that the Nrf2 pathway activation is a cellular mechanism underlying the beneficial effects of BBE-IP against intestinal inflammation induced by TNF-α.

## 3. Discussion

Several epidemiological studies have reported the beneficial effects of ACNs for human health in numerous diseases, including IBD [14,21]. These beneficial effects are mainly related to their antioxidant and anti-inflammatory activities. In particular, ACNs exert their effects both directly acting as antioxidants/free radical scavengers, as well as indirectly via the modulation of the redox-dependent transcriptional factors NF-κB and Nrf2 [21].

To date, most of the studies carried out to evaluate ACN activity used purified molecules at high concentrations, which may not be reached following gastrointestinal digestion [52]. Several studies, in fact, reported the poor availability of ACNs, especially due to the changes in pH and the metabolism they undergo during gastrointestinal digestion [26]. Generally, ACNs are stable in an acidic environment (pH 1–3) in which they exist as a flavilium cation, but at pH > 4, they adopt the forms of carbinol and chalcone that undergo further modifications producing phenolic acids [26,53]. Furthermore, in addition to undergoing conjugation reactions, the aglycones can be degraded in the intestinal lumen by enzymes present in the enterocytes and by the microbiota leading to the formation of phenolic acids and aldehydes [26,54]. In particular, the primary route of metabolism of C3G appeared to be through degradation to protocatechuic acid (PCA), followed by rapid methylation of the catechol group to form vanillic acid, and then sulfate or glucuronide conjugated and eliminated from the body [55]. Thus, following gastrointestinal digestion, the amount of ACN is reduced while that of phenolic acids, their by-products, increases, and these can be absorbed in the intestine through epithelial monocarboxylic acid transporters and then further metabolized in the kidneys or liver [12,55].

Therefore, with the aim of achieving a more realistic knowledge about the potential protective effect of ACN, an in vitro simulated gastrointestinal digestion of a purified and standardized bilberry and blackcurrant extract rich in ACN (BBE) was performed, followed by the evaluation of the modification of ACN composition and antioxidant activity. We further studied the in-situ effect of the intestinal phase of the simulated digestion on an in vitro model of intestinal inflammation by using differentiated Caco-2 cells exposed to TNF-α.

In vitro gastrointestinal digestion models are very advantageous systems for assessing the fate of a specific food or substance and the impact of the food matrix and food components on their stability and bioactivity. Our data have shown that ACN, subjected to in vitro simulated gastrointestinal digestion, are highly unstable molecules and susceptible to pH variations, as suggested by a huge reduction in RI compared to the sham-digested extract (Figure 1). In particular, a strong reduction in the RI was observed in oral and intestinal phases, and this suggests that a pH above 4 leads to the formation of colorless hemiketals and consequently to the opening of the C ring [26,56]. In the sample subjected to intestinal digestion, the possible development of their degradation products could also participate in the biological properties [26]. These findings are in agreement with those previously reported by Correa-Betanzo and coworkers [34], demonstrating that the ACN of the wild blueberry extract (*Vaccinium angustifolium*), subjected to in vitro simulated gastrointestinal digestion, underwent a strong reduction compared to the amount present in the undigested extract, with RI values in the intestinal phase extract of 17%. In another study conducted by Marhuenda and coworkers [33], evaluating the ACN stability by determining the total ACN content, they found a loss of 68.35% and 90.1% for blueberry and blackberry extracts, respectively, following in vitro simulated gastrointestinal digestion.

The high loss in the ACN content during gastrointestinal digestion is related to a reduction in the antioxidant power of BBE, as suggested by our FRAP test data (Figure 2) and in accordance with what has been observed in other studies [31,32,37]. However, the considerable reduction in ACN content following intestinal digestion vs. gastric one corresponded to double the antioxidant power than would be expected from the remaining ACN content, suggesting that in this case, the antioxidant capacity was not only due to the ACN content but probably to a synergistic effect between these compounds and the new metabolites formed.

In addition, the HPLC analysis also confirmed the high instability of the ACN following gastrointestinal digestion (Figure 3 and Table 1). In fact, of the 17 ACN present in the sham-digested extract, only six of them were found after gastrointestinal digestion, and a significant reduction was observed among those remaining compared to the amount present in the sham-digested extract. The presence of cyanidin derivatives and the disappearance of delphinidin derivatives were mainly determined, suggesting that the chemical structure (such as the number of hydroxyl groups) influences their stability. This reduction in the number and quantity of ACN in the intestinal phase extract is due to the nature and, therefore, the stability of the various ACN [38], as also reported in previous studies [30,41].

Finally, since the studies in the literature on in vitro models use concentrations difficult to reach at the target site, not considering the effects due to gastrointestinal digestion after oral administration, we tested the effect of the BBE extract rich in ACNs after simulated gastrointestinal digestion (BBE-IP) on an in vitro model of intestinal inflammation using Caco-2 cells. Our data highlight the anti-inflammatory effect of BBE-IP through the inhibition of the NF-κB pathway activated by TNF-α. In fact, we found reduced nuclear levels of NF-κB (p65) (Figure 4) and downregulation of IL-6 and IL-8 in cells pretreated with BBE-IP (Figure 5). These findings are in agreement with those previously reported by Le Phoung Nguyen and coworkers [57], reporting that an extract rich in ACN from *Prunus cerassus* inhibited the activation of the NF-κB inflammatory pathway and consequently reduced the levels of IL-8 and IL-6 pro-inflammatory cytokines in Caco-2 cells. This ability of ACN to inhibit the activation of this inflammatory pathway has also been highlighted in other studies conducted on similar experimental models using wild blueberry powder [58], grape seed and grape marc extract [43], digested ACN-rich extracts from root purple potato or purple carrot [44]. In addition, in our experimental conditions, BBE-IP was able to activate an antioxidant cellular adaptive response even at very low concentrations (0.75 μg/mL C3G eq./mL) by increasing the nuclear levels of Nrf2 (Figure 6) as well as the gene expression of NQO1 (Figure 7). In fact, the protective effect of ACN against IBD is not only attributable to the anti-inflammatory action but also to the activation of an antioxidant cell adaptive response mediated by the Nrf2 pathway [18,20,59,60]. Finally, the results herein obtained confirm that the protective effects exerted by the ACN could be mediated by the interaction with Nrf2 and NF-κB and further support the hypothesis of crosstalk between these two cell signaling pathways.

In conclusion, our findings help clarify the molecular mechanisms through which ACN exert positive effects on intestinal inflammation and suggest their possible protective role in the prevention or reduction in IBD development. Although ACNs are highly unstable molecules during gastrointestinal digestion and suffer a large loss because of this process, our results confirmed the anti-inflammatory and antioxidant activity of digested products rich in ACN, very likely also due to the formation of metabolites acting synergistically with their precursors. However, although a precise prediction of the in vivo bio-accessibility is partial since static models lack the simulation of accurate enzyme–substrate ratios, pH profiles, and transit times [61], and do not consider degradation in the intestinal lumen by enzymes present in the enterocytes and by the microbiota, fist-pass metabolism, and enterohepatic recycling, further studies are required to in vivo confirm the relevance of our data.

## 4. Materials and Methods

### 4.1. Reagents

Porcine α-amylase, porcine pepsin, porcine pancreatin, porcine bile, potassium chloride, potassium monobasic phosphate, sodium bicarbonate, magnesium chloride, ammonium carbonate, Folin-Ciocalteau reagent, ammonium acetate, and sodium carbonate were all purchased from Sigma Aldrich (UK). The primary antibodies, anti-NF-κB p65 and anti-Nrf2, were bought from Santa Cruz Biotechnology. The antibodies anti-Lamin B and secondary antibodies HRP-labeled goat anti-rabbit Ig were all purchased from Cell Signaling Technology. The E.Z.N.A. A Total RNA Kit was bought from OMEGA bio-tek (VWR). The Quanti-IT RNA assay and the Tumor Necrosis Factor-α (TNF-α) were purchased from Invitrogen GIBCO (Milan, Italy). All of the other reagents, unless otherwise specified, were acquired from Sigma-Aldrich (Milan, Italy).

### 4.2. In Vitro Simulated Gastrointestinal Digestion

**Figure 8:**
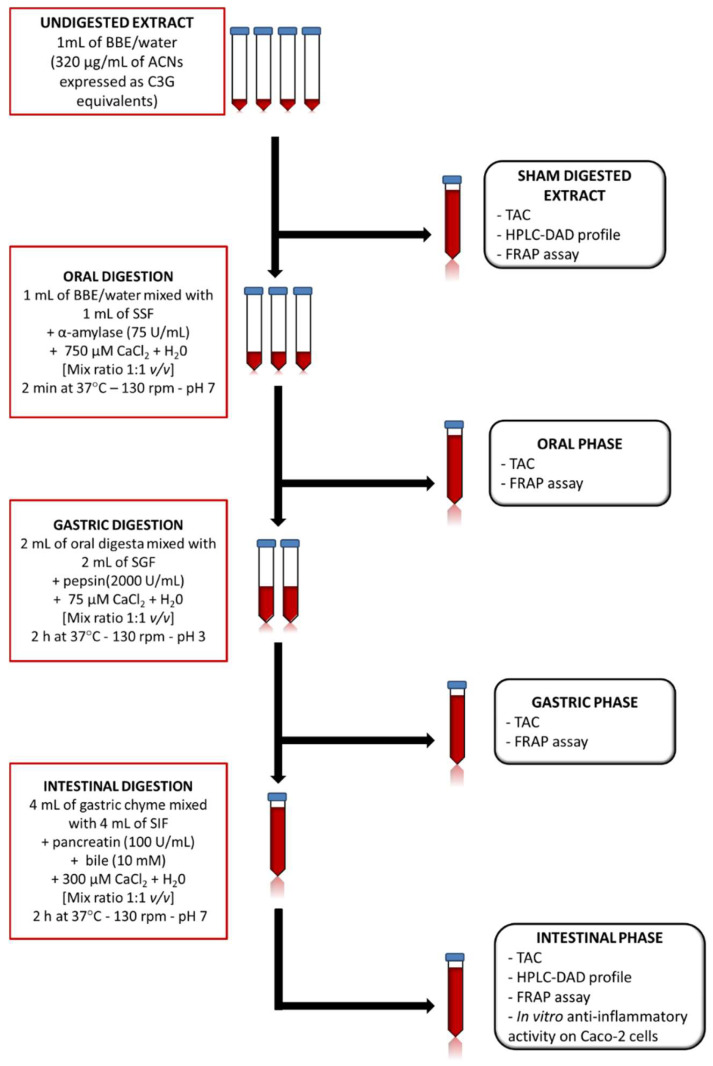
Schematic workflow of the in vitro simulated gastrointestinal digestion method.

The blackcurrant and bilberry extract (BBE) (Medox^®^; Biolink Group AS, Sandnes, Norway) used in the present study is an anthocyanin enriched dietary supplement, commercially available, consisting of 17 purified ACN (all glycosides of cyanidin, peonidin, delphinidin, petunidin, and malvidin) isolated from wild bilberries (*Vaccinium myrtillus*) and blackcurrant (*Ribes nigrum*). The in vitro gastrointestinal digestion of BBE was carried out following the method described by Minekus and coworkers [61]. The in vitro gastrointestinal digestion consists of three phases: oral, gastric, and intestinal (small intestinal) phase (Figure 8).

For all experiments of in vitro digestion, BBE was solubilized with water obtaining a concentration of 320 µg/mL of ACN expressed as C3G equivalents, as described below.

*Oral digestion*: 1 mL of BBE solution was incubated (in ratio 1:1, *v*/*v)* with a mixture containing salivary simulated fluid (SSF) (KCl 15.1 mM, KH_2_PO_4_ 3.7 mM, NaHCO_3_ 13.6 mM, MgCl_2_(H_2_O)_6_ 150 μM, (NH_4_)_2_CO_3_ 60 μM, pH = 7), salivary α-amylase (final conc. 75 U/mL), 750 μM CaCl_2_ and water for 2 min at 37 °C and in a continuous agitation (130 rpm) using a shaking water bath.

*Gastric digestion*: 2 mL of the remaining oral digesta were mixed (in ratio 1:1, *v*/*v)* with gastric simulated fluid (GSF) (KCl 6.9 mM, KH_2_PO_4_ 900 μM, NaHCO_3_ 25 mM, MgCl_2_(H_2_O)_6_ 100 μM, (NH_4_)_2_CO_3_ 500 μM, NaCl 47.2 mM), ultra-pure water and CaCl_2_ 75 μM (final conc.). HCl 1 M was used to adjust pH to 3. Then, the mixture was incubated at 37 °C for 2 h with porcine pepsin (final conc. 2000 U/mL) and freshly solubilized in ultra-pure water in a shaking water bath (130 rpm).

*Intestinal digestion*: 4 mL of the resulting gastric chyme were mixed (in ratio 1:1, *v*/*v)* with the intestinal simulated fluid (SIF) (KCl 6.8 mM, KH_2_PO_4_ 800 μM, NaHCO_3_ 85 mM, MgCl_2_(H_2_O)_6_ 330 μM, NaCl 38.4 mM), pure water and CaCl_2_ 300 μM (final conc.). NaOH 1 M was used to neutralize the pH (pH = 7), and immediately the mixture was incubated with bile (10 mM) and porcine pancreatin (100 TAME U/mL) for 2 h at 37 °C in a shaking water bath.

The final volume of the samples obtained from oral and gastric digestion or sham-digested was adjusted in parallel to 8 mL, corresponding to the final volume of intestinal digesta. In addition, the pH of all of the samples was adjusted with HCl 1 M up to pH 2 to keep ACNs stable, and the enzymatic activity was blocked by low temperature (4 °C). Finally, the samples were centrifuged at 5000 rpm for 10 min, and the supernatant was collected and stored at −80 °C until use.

### 4.3. Total Anthocyanins Content (TAC) and Recovery Index (RI)

The pH differential method described by the Association of Official Analytical Chemists (AOAC) [62] was used to evaluate the total ACN content. This method exploits the ability of ACN to change color in a reversible way according to the pH variation. At pH 1.0, the colored flavylium cation form prevails, while at pH 4.5, the colorless hemiketal is the predominant form. The samples of each digestion step or sham-digested were diluted with two buffers at different pH (0.025 M potassium chloride buffer pH 1 and 0.4 M sodium acetate buffer pH 4.5) and then incubated in the dark for 15 min. The absorbance of each sample diluted with the appropriate buffer was measured by UV/Vis spectrophotometer at 520 nm (maximum absorption peak of monomeric ACN) and 700 nm (wavelength in which ACN absorbance does not occur), as described by Giusti and Wrolstad [63]. The reading at 700 nm is essential to correct for possible light scattering in the event of a cloudy sample. The monomeric ACN pigment concentrations were expressed as C3G equivalents/mL and were calculated using the following formula:

(mg/mL) = (A MW DF)/ε*L

where A (Absorbance) = (A_520_ − A_700_) _pH1_ − (A_520_ − A_700_) _pH4_._5_, MW (C3G Molecular Weight) = 449.2, DF (Dilution Factor), 1 (Pathlength) = 1 cm, ε (C3G Molar Absorptivity) = 26,900 L/mol cm.

Finally, in order to determine the amount of ACN left after each step of the in vitro digestion process, the recovery index (RI) was calculated following the method reported by Ortega and coworkers [64].

% Recovery Index (RI) = (TAC digesta/TAC sham − digested BBE)*100

### 4.4. Ferric Reducing Antioxidant Power (FRAP) Assay

The antioxidant activity of sham-digested or digested BBE was carried out as described by Boussahel and coworkers [65]. This assay exploits the ability of non-enzymatic antioxidant compounds, such as polyphenols, to reduce, in acidic conditions, an uncolored complex Fe^3+^-TPTZ in blue complex Fe^2+^-TPTZ. Briefly, the digesta or sham-digested extracts were diluted in FRAP reagent, and after 4 min incubation at 20 °C, the absorbance was recorded at 593 nm. The sample concentration was assessed using the standard curve (FeSO_4_) equation, and the values were expressed as μmol FeSO_4_ equivalent/mL of digesta or sham-digested extract.

### 4.5. Anthocyanins Profile by HPLC-DAD

The ACN profiles of the undigested and intestinal digested samples were determined using an Agilent 1200 series HPLC system (Agilent Technologies, Santa Clara, CA, USA) equipped with a diode array detector (HPLC-DAD) according to Czank and coworkers [66]. Chromatographic separations were carried out on a Kinetex XB-C18 column (100 × 4.6 mm, 2.6 μm particle size). The mobile phase consisted of two solvents: formic acid in water (5:95, *v*/*v*) (solvent A) and formic acid in acetonitrile (5:95, *v*/*v*) (solvent B). A gradient elution program was performed as follows: 2.5% B, 0–15 min; 25% B, 15–20 min; 45% B, 20–25 min, and 100% B at 25−30 min. The flow rate was 1 mL/min. Samples were filtered through a 0.2-µm PTFE membrane disk filter, and 20 µL were injected into the column. Since ACNs show a characteristic and specific absorption peak at 520 nm deriving from the absorption of the benzopyran ring, UV-Vis spectra were recorded in the range of 200–600 nm, and chromatograms were acquired at 520 nm. To confirm the identified peaks, the retention time and UV absorption spectra of each compound were compared to those of anthocyanin standards.

### 4.6. Cell Culture and Treatments

Caco-2 human epithelial cells, obtained from American Tissue Culture Collection (ATCC), were cultured and differentiated in order to better reproduce the in vivo intestinal condition, as previously described [18,19]. All of the experiments were carried out on fully differentiated cells. The measurement of Trans-Epithelial Electrical Resistance (TEER) by using a Millicell-ERS Voltohmmeter (Millipore, Burlington, MA, USA) was performed to evaluate monolayer integrity and formation of tight junction (TJ). The experiments were conducted on monolayers with TEER values ≥ 600 Ω × cm^2^.

Fully differentiated Caco-2, prepared as described above, were pretreated or not for 24 h with different concentrations of the BBE intestinal phase (BBE-IP) (0.18, 0.37, 0.75, and 1.5 μg/mL, calculated with the pH differential method described above and expressed as C3G equivalents) added only to the apical compartment of the permeable filter support. At the end of the incubation time, the cells were rinsed twice with DPBS with calcium and magnesium at both the compartments and were exposed to TNF-α 50 ng/mL for 6 h, added in both the apical and the basolateral compartments of the transwell inserts.

### 4.7. Nuclear Proteins Extraction

After the appropriate treatments, nuclear proteins were extracted as previously described [67]. The nuclear fractions were stored at −80 °C until use. Nuclear protein concentration was evaluated by the Bradford colorimetric assay [68] using bovine serum albumin (BSA) as a standard.

### 4.8. Western Blot Analysis

For the immunoblotting analysis, 40 μg of protein were denatured in 4X SDS-PAGE reducing sample buffer and subjected to the SDS-PAGE on 12% acrylamide/bisacrylamide gels in order to evaluate NF-κB p65 and Nrf2. Quantitative analysis was performed by densitometry. Protein loading homogeneity was evidenced by Ponceau staining and housekeeping protein Lamin B.

### 4.9. Quantitative RT-PCR

The total cellular RNA was isolated using the E.Z.N.A. Total RNA Kit (OMEGA Bio-tek VWR, Invitrogen), quantified by the Quanti-iT^TM^ RNA assay kit QUBIT (Invitrogen, Milan, Italy), and reverse transcripted with M-MLV Reverse Transcriptase. Real-time PCR was performed on an ABI 7300 Real-Time PCR System with SYBR green chemistry (SYBR green JumpStart Taq Ready Mix—Sigma), employing primers already described [19,69,70]. The fold increase in mRNA expression was compared with the control cells (not pretreated and not exposed to TNF-α), corrected with the 18S rRNA reference gene, and determined using the 2^−ΔΔCt^ method [71].

### 4.10. Statistical Analysis

All of the experiments were carried out in triplicate and repeated three times. The results are expressed as mean ± SD from three independent experiments and statistically analyzed by a one-way ANOVA test, followed by Tukey’s HSD, using the statistically ezANOVA (https://people.cas.sc.edu/rorden/ezanova/index.html, accessed on 1 June 2022), differences between groups and treatments were considered significant for *p* < 0.05.

## Figures and Tables

**Table 1:** **Anthocyanins profile and stability of BBE before and after in vitro intestinal digestion, as determined by HPLC/DAD.** Data represent peak area (mAU*s) at 520 nm. RI% is the recovery index of each ACN in the intestinal phase vs. the initial content in sham-digested extract. Results are expressed as ± SD of three different experiments ^a^ *p* < 0.05 vs. respective ACN from sham-digested extract, ^b^ *p* < 0.05 vs. sham-digested extract.

Anthocyanins			Sham-Digested Extract	Gastro-Intestinal Extract	
Peak Number		Retention Time min	mAU*s	mAU*s	RI (%)
1	Del-3-gal	7.382	68.61 ± 4.85	-	-
2	Del-3-glu	9.082	125.74 ± 8.89	-	-
3	Cya-3-gal	10.163	52.30 ± 3.69	15.67 ± 1.11 ^a^	29.96
4	Del-3-Ara	10.776	54.36 ± 3.84	-	-
5	Del-3-rut	11.526	146.71 ± 10.37	-	-
6	Cya-3-glu	12.475	91.37 ± 6.46	22.65 ± 1.41 ^a^	24.79
7 + 8	Pet-3-gal + Cya-3-ara	13.813	66.11 ± 4.67	17.08 ± 1.21 ^a^	25.84
9	Cya-3-rut	15.132	170.49 ± 12.05	66.26 ± 3.55 ^a^	38.86
10	Pet-3-glu	15.602	59.73 ± 4.22	-	-
11	Peo-3-gal	16.352	10.75 ± 0.76	-	-
12	Pet-3-ara	16.897	12.86 ± 0.90	-	-
13	Peo-3-glu	18.018	23.56 ± 1.66	-	-
14	Mal-3-gal	18.353	15.56 ± 1.10	-	-
15	Peo-3-ara	18.688	7.25 ± 0.65	-	-
16	Mal-3-glu	19.075	54.20 ± 3.83	16.50 ± 1.02 ^a^	30.44
17	Mal-3-ara	19.478	9.59 ± 0.68	-	-
	TOTAL		969.19 ± 68.62	138.16 ± 8.30 ^b^	14.25

## Data Availability

The data that support the findings of this study are available on reasonable request from the corresponding author, A.S. (Antonella Saija).
